# Prevalence of multisite musculoskeletal symptoms: a French cross-sectional working population-based study

**DOI:** 10.1186/1471-2474-13-122

**Published:** 2012-07-20

**Authors:** Elsa Parot-Schinkel, Alexis Descatha, Catherine Ha, Audrey Petit, Annette Leclerc, Yves Roquelaure

**Affiliations:** 1 LUNAM Université, Université d’Angers, Laboratoire d’ergonomie et d’épidémiologie en santé au travail (LEEST), 49045, Angers, France; 2 CHU d’Angers, 49933, Angers, France; 3 Université de Versailles St-Quentin, UMRS 1018, Centre for Research in Epidemiology and Population Health, Population-Based Epidemiological Cohorts Research Platform, 94807, Villejuif, France; 4 Département santé travail, Institut de veille sanitaire, 94415, Saint-Maurice, France

## Abstract

**Background:**

The musculoskeletal disorders in working population represent one of the most worrying work-related health issues at the present time and although the very great majority of available data on the subject focus on musculoskeletal disorders defined by anatomical site, a growing number of studies indicate the low prevalence of disorders strictly confined to a specific anatomical site. The objective of this study was to describe the prevalence and characteristics of multisite musculoskeletal symptoms (multisite MS) in a large French working population.

**Methods:**

This study was performed on surveillance data of the cross-sectional survey (2002–2005) conducted by a network of occupational physicians in the working population of the Loire Valley region (from 20 to 59 years old). Data concerning MS were collected in the waiting room of the occupational physicians by means of the self-administrated standardized NORDIC questionnaire.

**Results:**

The study population comprised 3,710 workers (2,162 men (58%) and 1,548 women (42%)) with a mean age of 38.4 years (standard deviation: 10.4 years). The prevalence of MS during the past 12 months was 83.8% with 95% confidence interval of [82.8-85.3] for men and 83.9% [82.0-85.7] for women. The prevalence of subacute MS (lasting at least 30 days) over the past 12 months was 32.8% [30.9-34.8] for men and 37.3% [34.9-39.7] for women. Two-thirds of workers reported MS in more than one anatomical site and about 20% reported MS lasting at least 30 days in more than one anatomical site. The anatomical sites most frequently associated with other MS were the upper back, hip, elbow and neck. The majority of these multisite MS were widespread, involving at least two of the three anatomical regions (upper limb, axial region and lower limb).

**Conclusions:**

The frequency and extent of multisite MS reported by workers are considerable. Further research must be conducted in this field in order to provide a better understanding of the characteristics and determinants of these multisite MS.

## Background

All over the world, musculoskeletal disorders (MSDs) are responsible for considerable human, social and work-related burdens in terms of pain, distress at work, disability and quality of life. This major health issue has been recognized by the United Nations and the World Health Organization, which approved The Bone and Joint Decade 2000–2010 [1]. MSDs in working population are the leading cause of morbidity and work disability in the European Union [2,3] and represent one of the most worrying work-related health issues at the present time. According to Eurostat [4], the Statistical Office of the European Communities, MSDs are the most widespread and most costly work-related health problem in Europe, affecting about 45 million workers. A better understanding of the mechanisms responsible for the onset and progression of these disorders constitutes a major public health challenge in order to improve the prevention, management and prognosis of these disorders. For many years, research has therefore been largely devoted to risk factors and prognostic factors of MSDs demonstrating, regardless of the site studied, an increased risk related to cumulative biomechanical, psychosocial and organizational stresses.

The very great majority of the available data (descriptive, aetiological and prognostic) focus on MSDs defined by anatomical site. Recent studies support a more global approach to musculoskeletal disorders analysing the extent of musculoskeletal symptoms (MS) and especially the number of symptomatic anatomical sites rather than a particular site, either in the general population [5-17] or in the working population [18-24]. The results of these studies indicate the moderate prevalence of symptoms strictly confined to a specific anatomical site (estimated prevalence of 15 to 30% depending on the study) and the predominance of multisite MS (prevalence in the general population about 1/3 and 2/3 in the working population).

This characterization of MS based on the number of symptomatic sites regardless of the anatomical sites appears to be particularly suitable to study widespread pain according the American College of Rheumatology definition (WSP) [25,26]. However, this appears to be insufficient to describe less widespread multisite MS, as Picavet et al. clearly described that although involvement of several sites in the same region was very common, involvement of several sites located in distinct regions was also common [15]. It would be reasonable to suppose that when four anatomical sites are affected, the pathophysiology and prognosis may vary according to their regional distribution (symptoms affecting a single anatomical region or extending to more than one region). Croft challenged the conventional approach to MS defined by anatomical site by proposing a new and more relevant definition taking into account the extent of MS by region [27].

Although several teams have started to describe the profiles of these multisite MS [15,16,20,24] or 2-by-2 combinations corresponding to these multisite MS [18,19,21,24,28-31], very few studies on multisite MS and their corresponding profiles have been published. Many of these studies were also conducted on very specific populations [18,20,23,24,32,33] and/or small sample sizes [18,20,23,24,32].

The objective of this study, based on surveillance data in a large regional workforce, was to describe the prevalence and characteristics of multisite MS in a large population of workers.

## Methods

### Study design and population

The study was based on surveillance data collected by a network of occupational physicians (OPs) in the working population of the Loire Valley region (Central West France) [34]. The Pays de la Loire study was approved by both appropriate national committees : the *Comité consultatif sur le traitement de l’information en matière de recherché dans le domaine de la santé* (CCTIRS n°01-215) and the *Commission nationale de l’informatique et des libertés* (CNIL n°901 273).

The economic structure of this region (5% of the French working population) is diversified and similar to that of most French regions [35].

All French workers, including temporary and part-time workers, undergo a mandatory annual health examination by an OP in charge of the medical surveillance of a group of companies. Eighty-three OPs out of 460 (18% participation), representative of the region’s OPs, participated in the study. Each OP was trained by the investigators to randomly include workers undergoing a mandatory regularly-scheduled health examination between April 2002 and April 2005. The inclusion process followed a two-stage sampling procedure: first, the research team chose 15–45 half-days of scheduled examinations for each OP. Next, using random sampling tables, each OP selected 1 out of 10 workers from the schedule on the half-days of worker examinations considered. Among the regularly-scheduled health examination which had thus been selected (approximately 2.2% of the workers under surveillance by the 83 OPs), fewer than 10% of the selected workers were excluded (no shows, refusals, already included).

### Data

Data analysed in this article were collected by a questionnaire filled in by the workers during their annual visit. In particular, this questionnaire collected information on sociodemographic characteristics and on the presence and sites of MS. The presence and sites of MS were documented by a French version of the NORDIC questionnaire [36] including a mannequin with the anatomical sites considered, the standardized scale routinely used by occupational physicians for the detection of MS [37].

The following anatomical sites were studied: neck, shoulder/arm, elbow/forearm, hand/wrist, upper back, lower back, hip/thigh, knee/lower leg and ankle/foot.

Two definitions of MS were used in this article:

• presence of symptoms during the past 12 months by site;

• presence of symptoms lasting at least 30 days (prolonged) during the past 12 months by site.

For bilateral anatomical sites, MS were classed as present if they were reported on either or both sides of the body.

In results presented by anatomical region (axial, upper limb and lower limb), the neck was considered to be part of the upper limb. The presence of MS in an anatomical region for at least 30 days corresponded to presence in at least one site within the region for at least 30 days.

Multisite MS are defined by the presence of symptoms affecting more than one anatomical site on 9 studied sites.

### Statistical analysis

Classical statistical analyses were performed using SPSS software (v15; SPSS Inc., Chicago, IL, USA). All analyses were performed separately in men and women. The statistical unit was the individual, prevalence rates were calculated by dividing the number of subjects reporting symptoms (unilateral or bilateral) for the site of interest over the total number of responding subjects together with the 95% confidence intervals. Categorical data were compared with the Chi-square test or Fisher's exact test and a Mantel-Haenszel extension of the chi-square test for trend was used to test a linear trend. The limit of significance was 0.05.

## Results

The study population comprised 3,710 workers (2,162 men (58%), 1,548 women (42%), mean age: 38.4, SD: 10.4 years) out of 184,600 (2.0% sample) workers examined by the 83 OPs. Subjects mainly worked in service industries (59%), meat and manufacturing industries (34%), and more rarely in the construction (6%) and agriculture (1.5%) sectors. Men were mainly skilled and unskilled blue collar workers (56%), in intermediate occupations and technicians (25%), and managers and professionals (10%). Most women were low-grade white collar workers (52%), skilled and unskilled blue collar workers (24%), and in intermediate occupations and technicians (19%). Most workers, regardless of gender, presented a long service in the current job: more than ten years in 56% of cases, more than two years in 84% of cases and more than one year in 94% of cases.

### Prevalence of musculoskeletal symptoms (MS)

A total of 3,109 workers reported at least one MS affecting the limbs and/or spine during the past 12 months (1,811 men and 1,298 women). The corresponding prevalence rates were 83.8% with 95% confidence interval of [82.8-85.3] for men and 83.9% [82.0-85.7] for women.

At least one MS lasting at least 30 days during the past 12 months was reported by 1,287 workers (710 men and 577 women) with a prevalence of 32.8% [30.9-34.8] for men and 37.3% [34.9-39.7] for women (p = 0.005).

Prevalences of MS in the nine anatomical sites during the past 12 months are summarized in Table 1. The most frequent site was the lower back with MS reported by 59.3% [57.2-61.3] of men and 54.0% [51.5-56.5] of women (p = 0.0015) and with MS lasting at least 30 days reported by 16.6% [15.4-17.8] of all workers.

**Table 1:** Prevalence (%) and 95% confidence intervals (CI) of musculoskeletal symptoms during the past 12 months among men and women

**Symptoms: n (%) CI**	**Men (N = 2,162)**			**Women (N = 1,548)**		
Musculoskeletal symptoms:						
Neck symptoms**	725	(33.5)	31.5-35.5	747	(48.3)	45.8-50.7
Shoulder or upper arm symptoms*	735	(34.0)	32.0-36.0	601	(38.8)	36.4-41.3
Elbow or forearm symptoms	371	(17.2)	15.6-18.7	261	(16.9)	15.0-18.7
Wrist or hand symptoms**	468	(21.6)	19.9-23.4	463	(29.9)	27.6-32.2
Upper back symptoms**	451	(20.9)	19.1-22.6	503	(32.5)	30.2-34.8
Low back symptoms*	1281	(59.3)	57.2-61.3	836	(54.0)	51.5-56.5
Hip or thigh symptoms	360	(16.7)	15.1-18.2	278	(18.0)	16.0-19.9
Knee or lower leg symptoms	611	(28.3)	26.4-30.2	410	(26.5)	24.3-28.7
Ankle or foot symptoms	339	(15.7)	14.1-17.2	230	(14.9)	13.1-16.6
Musculoskeletal symptoms lasting at least 30 days:						
Neck symptoms**	142	(6.6)	5.5-7.6	185	(12.0)	10.3-13.6
Shoulder or upper arm symptoms**	197	(9.1)	7.9-10.3	202	(13.0)	11.4-14.7
Elbow or forearm symptoms	128	(5.9)	4.9-6.9	112	(7.2)	5.9-8.5
Wrist or hand symptoms**	130	(6.0)	5.0-7.0	154	(9.9)	8.5-11.4
Upper back symptoms**	121	(5.6)	4.6-6.6	174	(11.2)	9.7-12.8
Low back symptoms	352	(16.3)	14.7-17.8	264	(17.1)	15.2-18.9
Hip or thigh symptoms	102	(4.7)	3.8-5.6	91	(5.9)	4.7-7.1
Knee or lower leg symptoms	189	(8.7)	7.6-9.9	146	(9.4)	8.0-10.9
Ankle or foot symptoms	109	(5.0)	4.1-6.0	89	(5.7)	4.6-6.9

Significant differences between men and women: * p < 0.01, ** p < 0.0001.

The other most frequent sites of MS were the neck, shoulder and wrist in men and women, the upper back in women and the knee or lower leg in men with significant differences (for MS over the past 12 months and MS lasting at least 30 days over the past 12 months) between the two sexes (with the exception of the knee or lower leg) (Table 1).

### Prevalence of multisite MS

Two-thirds of workers reported the presence of MS involving more than one anatomical site (Table 2): 63.2% [61.1-65.2] of men and 68.3% [66.0-70.7] of women (p = 0.001).

**Table 2:** Prevalence (%) and 95% confidence intervals (CI) of multisite musculoskeletal symptoms (MS) during the past 12 months among men and women

**Multisite MS: n (%) CI**	**Men (N = 2,162)**			**Women (N = 1,548)**		
By genre*	1366	(63.2)	61.1-65.2	1058	(68.3)	66.0-70.7
By age group (test for linear trend) ^§‡^						
16-29 years	296	(57.6)	53.3-61.9	228	(63.2)	58.2-68.1
30-39 years	413	(63.5)	59.8-67.2	268	(62.5)	57.9-67.1
40-49 years	393	(64.2)	60.4-68.0	358	(73.5)	69.6-77.4
50-63 years	259	(68.3)	63.7-73.0	204	(75.6)	70.4-80.7
By occupational category^†^						
Managers and professionals	134	(63.8)	57.3-70.3	52	(66.7)	56.2-77.1
Associate professionals/technicians	337	(62.4)	58.3-66.5	195	(67.5)	62.1-72.9
Employees	113	(60.1)	53.1-67.1	523	(65.5)	62.2-68.8
Skilled and unskilled workers	773	(63.9)	61.2-66.6	285	(75.6)	71.3-79.9
By economic activity						
Service industries	664	(61.6)	58.7-64.5	726	(66.4)	63.6-69.2
Meat and manufacturing industries	559	(65.1)	61.9-68.3	293	(73.1)	68.7-77.4
Construction	123	(65.1)	58.3-71.9	16	(64.0)	45.2-82.8
Agriculture	19	(61.3)	44.1-78.4	19	(76.0)	59.3-92.7

Significant differences between men and women: * p < 0.01, ** p < 0.0001.

Significant differences among men: ^§^ p < 0.01, ^§§^ p < 0.0001.

Significant differences among women: ^†^ p < 0.01, ^‡^ p < 0.0001.

Slightly less than one third of workers reported MS involving four or more anatomical sites (27.3% of men and 34.0% of women), and 10% reported MS involving six or more sites (8.2% of men and 12.7% of women).

Slightly less than 20% of workers reported MS lasting at least 30 days in more than one anatomical site (Table 3): 17.1% [15.5-18.7] of men and 22.4% [20.3-24.4] of women (p < 0.0001) and 6.3% of workers reported MS lasting at least 30 days in four or more anatomical sites (4.9% of men and 8.3% of women), while 4.5% of women reported MS lasting at least 30 days in five or more anatomical sites (versus 2.0% of men).

**Table 3:** Prevalence (%) and 95% confidence intervals (CI) of multisite musculoskeletal symptoms (MS) lasting at least 30 days during the past 12 months among men and women

**Multisite MS: n (%) CI**	**Men (N = 2,162)**			**Women (N = 1,548)**		
By genre**	370	(17.1)	15.5-18.7	346	(22.4)	20.3-24.4
By age group (test for linear trend) ^§§‡^						
16-29 years	48	(9.3)	6.8-11.9	43	(11.9)	8.6-15.3
30-39 years	97	(14.9)	12.2-17.7	72	(16.8)	13.2-20.3
40-49 years	130	(21.2)	18.0-24.5	132	(27.1)	23.2-31.1
50-63 years	93	(24.5)	20.2-28.9	99	(36.7)	30.9-42.4
By occupational category^§^						
Managers and professionals	22	(10.5)	6.3-14.6	19	(24.4)	14.8-33.9
Associate professionals/technicians	86	(15.9)	12.8-19.0	54	(18.7)	14.2-23.2
Employees	30	(16.0)	10.7-21.2	170	(21.3)	18.5-24.1
Skilled and unskilled workers	232	(19.2)	17.0-21.4	102	(27.1)	22.6-31.5
By economic activity^†^						
Service industries	170	(15.8)	13.6-17.9	228	(20.9)	18.5-23.3
Meat and manufacturing industries	160	(18.6)	16.0-21.2	108	(27.0)	22.6-31.3
Construction	34	(18.0)	12.5-23.5	2	(8.0)	1.0-26.0^#^
Agriculture	5	(16.1)	3.2-29.1	6	(24.0)	7.3-40.7

Significant differences between men and women: * p < 0.01, ** p < 0.0001.

Significant differences among men: ^§^ p < 0.01, ^§§^ p < 0.0001.

Significant differences among women: ^†^ p < 0.01, ^‡^ p < 0.0001.

^*#*^ Estimation of CI with Fisher’s exact method (np or nq < 5).

**Figure 1:**
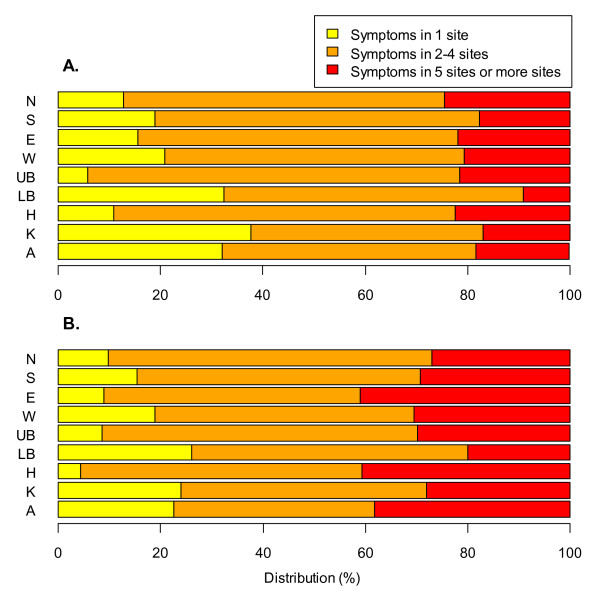
**Distribution of musculoskeletal symptoms lasting at least 30 days according to the number of symptoms.** These figures represent the distribution of musculoskeletal symptoms lasting at least 30 days during the past 12 months according to the number of declared symptoms among men (figure **A**) and women (figure **B**): Neck symptoms (N), Shoulder or upper arm symptoms (S), Elbow or forearm symptoms (E), Wrist or hand symptoms (W), Upper back symptoms (UB), Low back symptoms (LB), Hip or thigh symptoms (H), Knee or lower leg symptoms (K), Ankle or foot symptoms (A).

The prevalence of MS affecting two to four anatomical sites was three to twelve times more common than prevalence of MS affecting only one site in workers who reported musculoskeletal symptoms at a given anatomical site whatever it is (Figure 1).

More than 80% of MS lasting at least 30 days involving the upper back, hip, neck and elbow were associated with other MS (multisite MS). MS lasting at least 30 days involving the knee or lower leg, lower back and ankles were more frequently isolated (1/4 to 1/3 of cases).

The prevalence of multisite MS increased slightly but significantly with increasing age (test for linear trend: p = 0.001 for the men and p < 0.0001 for the women, Table 2). For the prevalence of multisite MS lasting at least 30 days, we see a significant increase with increasing age: slight for the men (test for linear trend: p < 0.0001, Table 3) but more marked for the women (test for linear trend: p < 0.0001, Table 3).

For the women, the prevalence of multisite MS was associated with the occupational category (p = 0.006, Table 2), we see a prevalence more raised for skilled and unskilled workers versus associate professionals and technicians.

The prevalence of multisite MS lasting at least 30 days was associated with the occupational category for the men and women (Table 3) with, for the men, a slightly lower prevalence for the managers and professionals (p = 0.013) and, for the women, a higher prevalence for skilled and unskilled workers (p = 0.052). Furthermore, for the women, we also observe a significant association between prevalence of multisite MS lasting at least 30 days and the activity sector with a higher prevalence for the meat and manufacturing industries versus the service industries (p = 0.026, Table 3).

### MS by anatomical region

The anatomical regions most frequently involved were the axial and upper limb regions with a prevalence of MS lasting at least 30 days over the past 12 months of 18 to 23% (Table 4).

**Table 4:** Prevalence (%) and 95% confidence intervals (CI) of musculoskeletal symptoms in various anatomical regions during the past 12 months among men and women

**Symptoms: n (%) CI**	**Men (N = 2,162)**			**Women (N = 1,548)**		
Musculoskeletal symptoms:						
Axial	1395	(64.5)	62.5-66.5	996	(64.3)	62.0-66.7
Upper limb (with neck)	1310	(60.6)	58.5-62.7	1067	(68.9)	66.6-71.2
Lower limb	942	(43.6)	41.5-45.7	623	(40.2)	37.8-42.7
Musculoskeletal symptoms lasting at least 30 days:						
Axial	383	(17.7)	16.1-19.3	335	(21.6)	19.6-23.7
Upper limb (with neck)	378	(17.5)	15.9-19.1	362	(23.4)	21.3-25.5
Lower limb	322	(14.9)	13.4-16.4	229	(14.8)	13.0-16.6

About one quarter of subjects (Table 5) reported the presence of MS involving a single anatomical region during the past 12 months (25.7% of men and 21.9% of women), usually involving axial regions in men (45.4% of symptoms localized to one region) and the upper limb in women (54.3% of symptoms localized to one region).

**Table 5:** Numbers of anatomical regions with musculoskeletal symptoms during the past 12 months among men and women

**Symptoms: n (%) CI**	**Men (N = 2,162)**			**Women (N = 1,548)**		
No region:	351	(16.2)	14.7-17.8	250	(16.1)	14.3-18.0
One region:	555	(25.7)	23.8-27.5	339	(21.9)	19.8-24.0
Axial	252	(11.7)	10.3-13.0	121	(7.8)	6.5-9.2
Upper limb (with neck)	211	(9.8)	8.5-11.0	184	(11.9)	10.3-13.5
Lower limb	92	(4.3)	3.4-5.1	34	(2.2)	1.5-2.9
Two regions:	676	(31.3)	29.3-33.2	530	(34.2)	31.9-36.6
Axial and upper limb	406	(18.8)	17.1-20.4	370	(23.9)	21.8-26.0
Axial and lower limb	157	(7.3)	6.2-8.4	76	(4.9)	3.8-6.0
Upper and lower limb	113	(5.2)	4.3-6.2	84	(5.4)	4.3-6.6
Three regions:	580	(26.8)	25.0-28.7	429	(27.7)	25.5-29.9

One-third of subjects (31.3% of men and 34.2% of women) reported the presence of MS involving two anatomical regions during the past 12 months (axial and upper limb for 2/3 of them) and 27.2% reported disorders involving the three anatomical regions studied: axial, upper limb and lower limb.

Nine to 12% of subjects reported the presence of MS in two anatomical regions for at least 30 days (Table 6) with a predominance of symptoms affecting the axial and upper limb regions (51.3% of symptoms localized to two regions).

**Table 6:** Numbers of anatomical regions of musculoskeletal symptoms lasting at least 30 days during the past 12 months among men and women

**Symptoms: n (%) CI**	**Men (N = 2,162)**			**Women (N = 1,548)**		
No region:	1452	(67.2)	65.2-69.1	971	(62.7)	60.3-65.1
One region:	425	(19.7)	18.0-21.3	308	(19.9)	17.9-21.9
Axial	148	(6.8)	5.8-7.9	101	(6.5)	5.3-7.8
Upper limb (with neck)	149	(6.9)	5.8-8.0	140	(9.0)	7.6-10.5
Lower limb	128	(5.9)	4.9-6.9	67	(4.3)	3.3-5.3
Two regions:	197	(9.1)	7.9-10.3	189	(12.2)	10.6-13.8
Axial and upper limb	91	(4.2)	3.4-5.1	107	(6.9)	5.6-8.2
Axial and lower limb	56	(2.6)	1.9-3.3	47	(3.0)	2.2-3.9
Upper and lower limb	50	(2.3)	1.7-2.9	35	(2.3)	1.5-3.0
Three regions:	88	(4.1)	3.2-4.9	80	(5.2)	4.1-6.3

More than 90% of multisite MS concerned two or three anatomical regions (91.9% for men and 90.6% for women).

Almost 80% of multisite MS lasting at least 30 days involved two or three anatomical regions (77.0% for men and 77.7% for women).

## Discussion

### Main results

This study presented analyses of the prevalence of multisite MS over a 12-month period in a general population of workers and described both the type and extent of other associated MS.

The main results of this study are:

• The frequency and extent of multisite MS were considerable in this population (2/3 with multisite MS with more than 90% of these multisite MS involving more than one anatomical region);

• Although multisite MS were significantly more frequent in women (68.3%), they were also very frequent in men (63.2%);

• The prevalence of multisite MS lasting more than 30 days was very high (17.1% men and 22.4% of women) and these symptoms were widespread (80% of these multisite MS involved more than one anatomical region).

### Methodological considerations

One of the strong points of this study is the large sample size (3,710 workers) and the representativity of the study population. The fact that all workers in France, including part-time or temporary workers, are submitted to an annual health check-up by an occupational physician in charge of the medical surveillance of a group of companies confirms that the recruitment of this study, based on a network of occupational physicians, is relevant to study the target population although farmers and self-employed workers, rarely followed by occupational physicians, would be underrepresented in this study. The representativity of the study sample compared to the population of the region and to the French population has already been detailed in a previous article [34]: Comparison of their socio-economic status with the last available French census (1999, http://www.insee.fr), the distribution of occupations showed no major differences for either gender with the regional workforce, except for the few occupations not surveyed by OPs (e.g., shopkeepers and self-employed workers).

The use of a self-administered questionnaire introduces a reporting bias inherent to this type of data collection leading probably to an over-estimation of recent and more serious musculoskeletal symptoms [38]. Furthermore, some individuals might have a tendency to report any symptoms, whereas others not report similar symptoms [39]. However, we have collected no information on the personality traits which could influence reporting patterns of symptoms. The standardized Nordic questionnaire [38] or derived questionnaires are commonly used in epidemiological studies on musculoskeletal disorders in the workplace or in the general population. The sensitivity and repeatability of this questionnaire are good and this questionnaire is likely to have a high utility in screening and surveillance [40-46]. The French version of this questionnaire [47] has a good sensitivity (100%) and moderate specificity (51%) in comparison with the physical examination of the upper limbs, according to the study of Descatha and al [48].

Lastly, this cross-sectional study cannot provide any information about the chronology and course of the symptoms described.

### Prevalence of MS

Estimated prevalences of MS reported in the literature vary considerably from one study to another, as they are highly dependent on the definition of musculoskeletal symptoms (types of symptoms, duration of symptoms and exposure period considered), the populations interviewed and the context of the study.

However, the results of this study are fairly concordant with those reported in the literature and the general knowledge on this subject. The prevalence of MS observed in this study (about 84%) is similar to the prevalence of 87% reported in several similar studies [11,13,20].

The results concerning the prevalence of MS by anatomical site over a 12-month period are also globally consistent with published data.

In a review of the literature [49], the prevalence of low back symptoms over a 12-month period was between 39 and 67%. The prevalence of 57% observed in the present study was therefore perfectly consistent with this range, as well as the estimations published in other studies [15,21,23,24]. The prevalence of MS of the upper back (26%) is also concordant with data of the literature (prevalences ranging from 6 to 33% [15,22,24]), as are the prevalences of MS of the elbow (17%, 6 to 21% in the literature [15,22-24]), hand (25%, 8 to 38% in the literature [15,18,22-24]) and hip (17%, 6 to 32% in the literature [15,22,24]).

Published data on the prevalence of MS over a 12-month period in other sites are more heterogeneous [15,18,19,22-24]. However, the estimated prevalences reported in the present study are consistent with published data, but are situated in the low range for the ankles or feet (15% in our study and 7 to 27% in the literature) and in the high range for the neck (40% in our study and 17 to 48% in the literature), shoulder (36% in our study and 19 to 39% in the literature) and knee or lower leg (28% in our study and 11 to 26% in the literature).

### Prevalence of multisite MS

The multisite MS described in this study were slightly more frequent and more extensive than those reported in the general population (50% of multisite MS and only 20.6% with MS in 4 or more sites) in the study by Picavet et al. [15]. When we compare the prevalence of multisite MS by sex and age group, we observe in our study in working population that the prevalences were on average twice as high that those observed in the study of Picavet in general population. In contrast, Kamaleri [11-14] reported more frequent and more extensive multisite MS in a general population cohort (75.4% of multisite MS and 37.5% with MS in 5 or more sites). However, Kamaleri et al. studied an additional anatomical site, the head, for which more than 30% of the population reported symptoms. This can probably explain the higher prevalence reported by Kamaleri and al. This anatomical site was not taken into account in this study, as head symptoms do not constitute a work-related MS.

High prevalences of multisite musculoskeletal pain are commonly found in many countries, but the precise comparison of prevalences of musculoskeletal pain in France and other high income countries is difficult due to the variability of the methods used. However, the results of the World Mental Health Surveys (WMHS) of multiple pains (2 or more sites with pain problems among the following 4 ones: back/neck pain, headaches, arthritis, other pain) in the general population are globally comparable for France and other countries [50]. These results are not comparable because the health problems taken into account by both studies are not strictly identical. Despite this limit, this result of the WMHS illustrate that the problem of the painful symptoms is globally comparable in the French population than in other high income countries.

Our data concerning the number of anatomical sites of MS differ from those reported by some studies conducted in working populations [18,20,23], as these studies targeted specific populations and only considered a limited number of sites (4 to 7 sites).

Yeung [24] reported similar frequencies of multisite MS in men workers (63.4%) to those reported in this study (63.9% for men workers), but, as in the study by Kamaleri, these symptoms were more widespread (22.7% with MS in 5 or more sites).

Although Haukka [20] only studied pain experienced during the past three months, he reported similar prevalence of MS (73% among female kitchen workers versus 75.6% for female workers in this study) and similar regional distribution of MS to that observed in this study.

### Prevalence of MS lasting at least 30 days

The estimated prevalences of MS lasting at least 30 days presented in this study are concordant with published data [18,51,52] and clearly confirm the importance of these subacute or chronic symptoms.

Multisite MS lasting at least 30 days among nurses, office workers and postal clerks in Crete were more frequent (1/3 versus, in our study, 15.8% for men and 20.9% for women in the service industries), but less widespread (only 4% with MS lasting at least 30 days in 4 or more sites), in the study by Solidaki [23].

In our study, MS lasting at least 30 days involving the knee or lower leg and lower back were often isolated. Conversely, MS lasting at least 30 days involving the upper back, hip and elbow, relatively uncommon in our study, were usually associated with other MS. This observation underlines that multisite MS do not necessarily correspond to the most frequent MS and suggests the existence of anatomical associations specific to multisite MS.

### Comparison between men and women

In this study, as in several previously published studies [21,23,33,53], 12-month prevalences were significantly higher in women for MS of the neck (+15%) and wrist (+8%). Twelve-month prevalences were also significantly higher in women than men for MS of the upper back (+11%) and shoulder (+5%), but were significantly lower for the lower back (−5%).

Significant differences, relatively moderate (4 to 6%), were also observed for MS lasting at least 30 days (with the exception of the lower back).

These findings are consistent with the observed differences in the prevalence of multisite MS: multisite MS were significantly more frequent in women (+5%), in agreement with the literature. This could reflect not only a higher tendency in women than men to report pain at multiple sites [54], but also a higher burden of disease among women [55]. The sites mostly frequently involved in women (neck, shoulder, wrist and upper back) also corresponded to the sites most frequently associated with others MS (i.e. multisite MS). On the other hand, MS of the lower back, less frequent in women, often corresponded to localized MS.

Nevertheless, multisite MS were also reported by a considerable proportion of men, including widespread MS (MS involving 5 or more sites).

Recent studies have specifically investigated differences between men and women [56,57]. Messing et al. demonstrated that gender was an independent risk factor for neck and lower limb pain even after adjustment for all of the identified personal and work-related risk factors. The proposed explanations for the impact of gender included different exposures and working conditions (even within the same type of job), an interaction between gender and personal factors, as well as biological and psychological differences. Silverstein et al. also reported higher prevalence rates of declared MS in women, but few differences in terms of diagnosed musculoskeletal disorders. Furthermore, in this last study, independent personal risk factors associated with MS of the wrist were more advanced age, presence of comorbidities and a high body mass index for women, while only more advanced age was an independent risk factor for men.

### Perspectives

Several studies have demonstrated a poorer state of health [11,30,58] associated with these multisite MS, especially in terms of sleep [11,58], comorbidities (other MS or vascular diseases) [59], functional capacity [12,15,17,58,60] and quality of life [30,60], and a poorer occupational prognosis [14,22,61,62]. The risk associated with these multisite symptoms increases with the number of sites affected, even after adjustment for the other identified risk factors [11-14,22].

The presence of regional or widespread MS has also been reported to be significantly correlated with excess mortality compared to subjects not experiencing MS, with an excess mortality of about 20% for regional pain (excess cancer mortality) and 30% for WSP (excess cancer and cardiovascular mortality), after adjustment for age, gender and ethnic group [63].

In this study, the presence of multisite MS was associated with the female gender and advanced age. However, the whole working population was concerned since the prevalence of the multisite MS lasting at least 30 days for the younger age group was not negligible (9% in men and 12% in women). The prevalence of multisite symptoms was little influenced by occupational categories and activity sector. So, these results do not allow the identification of specific risk groups to target future interventions of prevention.

## Conclusions

This study confirms the importance of multisite MS, including symptoms lasting at least 30 days. In view of the poor prognosis associated with these multisite MS, further research must be conducted on this subject in order to more clearly identify the various profiles of multisite MS and their determinants.

## Competing interests

None of the authors has any conflicts of interest to declare.

## Authors' contributions

Parot-Schinkel performed the analyses, participated to data interpretation, wrote the main part of the first version of the manuscript and made revisions after comments from the other authors. Descatha participated to data interpretation, to writing the first version of the manuscript and to comment the different versions of the manuscript. Ha and Leclerc participated to design the study, to data interpretation, and to comment the manuscript. Petit participated to data interpretation and to comment the different versions of the manuscript. Roquelaure designed and conducted the study, participated to data interpretation, and to comment the manuscript.

All authors read and approved the final manuscript

## Authors’ information

The authors are members of research units in occupational health and A Descatha, Y Roquelaure and A Leclerc are members of the Musculoskeletal Committee of the International Commission of Occupational Health (ICOH), and the French Language Research group on MSD.

## Pre-publication history

The pre-publication history for this paper can be accessed here:

http://www.biomedcentral.com/1471-2474/13/122/prepub

## References

[B1] Woolf AD The bone and joint decade 2000–2010 Ann Rheum Dis 2000 59 81 82 10.1136/ard.59.2.81 10666159 PMC1753078

[B2] European Agency for Safety and Health at Work Work-related neck and upperlimb musculoskeletal disorders 1999 Luxembourg: Office for Official Publications of the European Communities

[B3] European Foundation for the Improvement of Living and Working Conditions Third European Survey on Working Conditions 2000 2001 Luxembourg: Office for Official Publications of the European Communities

[B4] Eurostat Work and health in the EU: a statistical portrait. Data 1994–2002 2004 Luxembourg: Office for official publications of the European communities

[B5] Allison TR Symmons DPM Brammah T Haynes P Rogers A Roxby M Urwin M Musculoskeletal pain is more generalised among people from ethnic minorities than among white people in Greater Manchester Ann Rheum Dis 2002 61 151 156 10.1136/ard.61.2.151 11796402 PMC1753985

[B6] Carnes D Parsons S Ashby D Breen A Foster NE Pincus T Vogel S Underwood M Chronic musculoskeletal pain rarely presents in a single body site: results from a UK population study Rheumatology (Oxford) 2007 46 1168 1170 10.1093/rheumatology/kem118 17488750

[B7] Davies H Crombie I Macrae W Where does it hurt? Describing the body locations of chronic pain Eur J Pain 1998 2 69 80 10.1016/S1090-3801(98)90048-9 10700303

[B8] Gerdle B Björk J Cöster L Henriksson K Henriksson C Bengtsson A Prevalence of widespread pain and associations with work status: a population study BMC Musculoskelet Disord 2008 9 102 10.1186/1471-2474-9-102 18627605 PMC2488345

[B9] Harkness EF Macfarlane GJ Silman AJ McBeth J Is musculoskeletal pain more common now than 40 years ago?: Two population-based cross-sectional studies Rheumatology (Oxford) 2005 44 890 895 10.1093/rheumatology/keh599 15784630

[B10] Jordan KP Kadam UT Hayward R Porcheret M Young C Croft P Annual consultation prevalence of regional musculoskeletal problems in primary care: an observational study BMC Musculoskelet Disord 2010 11 144 10.1186/1471-2474-11-144 20598124 PMC2903510

[B11] Kamaleri Y Natvig B Ihlebaek CM Benth JS Bruusgaard D Number of pain sites is associated with demographic, lifestyle, and health-related factors in the general population Eur J Pain 2008 12 742 748 10.1016/j.ejpain.2007.11.005 18160318

[B12] Kamaleri Y Natvig B Ihlebaek CM Bruusgaard D Localized or widespread musculoskeletal pain: does it matter? Pain 2008 138 41 46 10.1016/j.pain.2007.11.002 18077092

[B13] Kamaleri Y Natvig B Ihlebaek CM Benth JS Bruusgaard D Change in the number of musculoskeletal pain sites: A 14-year prospective study Pain 2009 141 25 30 10.1016/j.pain.2008.09.013 18977088

[B14] Kamaleri Y Natvig B Ihlebaek CM Bruusgaard D Does the number of musculoskeletal pain sites predict work disability? A 14-year prospective study Eur J Pain 2009 13 426 430 10.1016/j.ejpain.2008.05.009 18599328

[B15] Picavet HSJ Schouten JSAG Musculoskeletal pain in the Netherlands: prevalences, consequences and risk groups, the DMC(3)-study Pain 2003 102 167 178 10.1016/s0304-3959(02)00372-x 12620608

[B16] Schmidt CO Baumeister SE Simple patterns behind complex spatial pain reporting? Assessing a classification of multisite pain reporting in the general population Pain 2007 133 174 182 10.1016/j.pain.2007.04.022 17570587

[B17] Urwin M Symmons D Allison T Brammah T Busby H Roxby M Simmons A Williams G Estimating the burden of musculoskeletal disorders in the community: the comparative prevalence of symptoms at different anatomical sites, and the relation to social deprivation Ann Rheum Dis 1998 57 649 655 10.1136/ard.57.11.649 9924205 PMC1752494

[B18] Alexopoulos EC Stathi I-C Charizani F Prevalence of musculoskeletal disorders in dentists BMC Musculoskelet Disord 2004 5 16 10.1186/1471-2474-5-16 15189564 PMC441388

[B19] Alexopoulos EC Tanagra D Konstantinou E Burdorf A Musculoskeletal disorders in shipyard industry: prevalence, health care use, and absenteeism BMC Musculoskelet Disord 2006 7 88 10.1186/1471-2474-7-88 17125504 PMC1676002

[B20] Haukka E Leino-Arjas P Solovieva S Ranta R Viikari-Juntura E Riihimäki H Co-occurrence of musculoskeletal pain among female kitchen workers Int Arch Occup Environ Health 2006 80 141 148 10.1007/s00420-006-0113-8 16688464

[B21] Kääriä S Solovieva S Leino-Arjas P Associations of low back pain with neck pain: a study of industrial employees with 5-, 10-, and 28-year follow-ups Eur J Pain 2009 13 406 411 10.1016/j.ejpain.2008.05.006 18571952

[B22] Morken T Riise T Moen B Hauge SHV Holien S Langedrag A Pedersen S Saue ILL Seljebø GM Thoppil V Low back pain and widespread pain predict sickness absence among industrial workers BMC Musculoskelet Disord 2003 4 21 10.1186/1471-2474-4-21 12956891 PMC200978

[B23] Solidaki E Chatzi L Bitsios P Markatzi I Plana E Castro F Palmer K Coggon D Kogevinas M Work-related and psychological determinants of multisite musculoskeletal pain Scand J Work Environ Health 2010 36 54 61 10.5271/sjweh.2884 20011982 PMC3242043

[B24] Yeung SS Genaidy A Deddens J Alhemood A Leung PC Prevalence of musculoskeletal symptoms in single and multiple body regions and effects of perceived risk of injury among manual handling workers Spine 2002 27 2166 2172 10.1097/00007632-200210010-00017 12394933

[B25] Lindell L Bergman S Petersson IF Jacobsson LT Herrström P Prevalence of fibromyalgia and chronic widespread pain Scand J Prim Health Care 2000 18 149 153 10.1080/028134300453340 11097099

[B26] Neumann L Buskila D Epidemiology of fibromyalgia Curr Pain Headache Rep 2003 7 362 368 10.1007/s11916-003-0035-z 12946289

[B27] Croft P Dunn KM Von Korff M Chronic pain syndromes: you can’t have one without another Pain 2007 131 237 238 10.1016/j.pain.2007.07.013 17728065

[B28] Gummesson C Atroshi I Ekdahl C Johnsson R Ornstein E Chronic upper extremity pain and co-occurring symptoms in a general population Arthritis Rheum 2003 49 697 702 10.1002/art.11386 14558056

[B29] Hagen EM Svensen E Eriksen HR Ihlebaek CM Ursin H Comorbid subjective health complaints in low back pain Spine 2006 31 1491 1495 10.1097/01.brs.0000219947.71168.08 16741460

[B30] IJzelenberg W Burdorf A Impact of musculoskeletal co-morbidity of neck and upper extremities on healthcare utilisation and sickness absence for low back pain Occup Environ Med 2004 61 806 810 10.1136/oem.2003.011635 15377765 PMC1740669

[B31] Macfarlane GJ Hunt IM Silman AJ Role of mechanical and psychosocial factors in the onset of forearm pain: prospective population based study BMJ 2000 321 676 679 10.1136/bmj.321.7262.676 10987773 PMC27483

[B32] Molano SM Burdorf A Elders LA Factors associated with medical care-seeking due to low-back pain in scaffolders Am J Ind Med 2001 40 275 281 10.1002/ajim.1099 11598974

[B33] Saastamoinen P Leino-Arjas P Laaksonen M Martikainen P Lahelma E Pain and health related functioning among employees J Epidemiol Community Health 2006 60 793 798 10.1136/jech.2005.043976 16905725 PMC2566029

[B34] Roquelaure Y Ha C Leclerc A Touranchet A Sauteron M Melchior M Imbernon E Goldberg M Epidemiologic surveillance of upper-extremity musculoskeletal disorders in the working population Arthritis Rheum 2006 55 765 778 10.1002/art.22222 17013824

[B35] Ha C Roquelaure Y Leclerc A Touranchet A Goldberg M Imbernon E The French Musculoskeletal Disorders Surveillance Program: Pays de la Loire network Occup Environ Med 2009 66 471 479 10.1136/oem.2008.042812 19269944 PMC2693672

[B36] Hagberg M Silverstein B Wells R Work related musculoskeletal disorders (WMSDs): a reference book for prevention 1995 London: Taylor & Francis

[B37] Descatha A Roquelaure Y Chastang J-F Evanoff B Cyr D Leclerc A Work, a prognosis factor for upper extremity musculoskeletal disorders? Occup Environ Med 2009 66 351 352 10.1136/oem.2008.042630 19376942 PMC2705760

[B38] Kuorinka I Jonsson B Kilbom A Vinterberg H Biering-Sørensen F Andersson G Jørgensen K Standardised Nordic questionnaires for the analysis of musculoskeletal symptoms Appl Ergon 1987 18 233 237 10.1016/0003-6870(87)90010-X 15676628

[B39] Tschudi-Madsen H Kjeldsberg M Natvig B Ihlebaek C Dalen I Kamaleri Y Straand J Bruusgaard D A strong association between non-musculoskeletal symptoms and musculoskeletal pain symptoms: results from a population study BMC Musculoskelet Disord 2011 12 285 10.1186/1471-2474-12-285 22176611 PMC3310803

[B40] Dickinson CE Campion K Foster AF Newman SJ O’Rourke AM Thomas PG Questionnaire development: an examination of the Nordic Musculoskeletal questionnaire Appl Ergon 1992 23 197 201 10.1016/0003-6870(92)90225-K 15676868

[B41] Ohlsson K Attewell RG Johnsson B Ahlm A Skerfving S An assessment of neck and upper extremity disorders by questionnaire and clinical examination Ergonomics 1994 37 891 897 10.1080/00140139408963698 8206057

[B42] Baron S Hales T Hurrell J Evaluation of symptom surveys for occupational musculoskeletal disorders Am J Ind Med 1996 29 609 617 10.1002/(SICI)1097-0274(199606)29:6<609::AID-AJIM5>3.0.CO;2-E 8773721

[B43] Silverstein BA Stetson DS Keyserling WM Fine LJ Work-related musculoskeletal disorders: comparison of data sources for surveillance Am J Ind Med 1997 31 600 608 10.1002/(SICI)1097-0274(199705)31:5<600::AID-AJIM15>3.0.CO;2-2 9099363

[B44] Palmer K Smith G Kellingray S Cooper C Repeatability and validity of an upper limb and neck discomfort questionnaire: the utility of the standardized Nordic questionnaire Occup Med (Lond) 1999 49 171 175 10.1093/occmed/49.3.171 10451598

[B45] Nordlund A Ekberg K Self reported musculoskeletal symptoms in the neck/shoulders and/or arms and general health (SF-36): eight year follow up of a case–control study Occup Environ Med 2004 61 e11 10.1136/oem.2002.005249 14985528 PMC1740735

[B46] Dawson AP Steele EJ Hodges PW Stewart S Development and test-retest reliability of an extended version of the Nordic Musculoskeletal Questionnaire (NMQ-E): a screening instrument for musculoskeletal pain J Pain 2009 10 517 526 10.1016/j.jpain.2008.11.008 19345154

[B47] Kuorinka I Jonsson B Kilbom A Vinterberg H Biering-Sorensen F Andersson G Analyse des problèmes de l’appareil locomoteur: questionnaire scandinave DMT 1994 58 167 170

[B48] Descatha A Roquelaure Y Chastang JF Evanoff B Melchior M Mariot C Ha C Imbernon E Goldberg M Leclerc A Validity of Nordic-style questionnaires in the surveillance of upper-limb work-related musculoskeletal disorders Scand J Work Environ Health 2007 33 58 65 10.5271/sjweh.1065 17353966 PMC2980505

[B49] McBeth J Jones K Epidemiology of chronic musculoskeletal pain Best Pract Res Clin Rheumatol 2007 21 403 425 10.1016/j.berh.2007.03.003 17602991

[B50] Gureje O Von Korff M Kola L Demyttenaere K He Y Posada-Villa J Lepine JP Angermeyer MC Levinson D de Girolamo G Iwata N Karam A Guimaraes Borges GL de Graaf R Browne MO Stein DJ Haro JM Bromet EJ Kessler RC Alonso J The relation between multiple pains and mental disorders: results from the World Mental Health Surveys Pain 2008 135 82 91 10.1016/j.pain.2007.05.005 17570586

[B51] Leclerc A Gourmelen J Chastang J-F Plouvier S Niedhammer I Lanoë J-L Level of education and back pain in France: the role of demographic, lifestyle and physical work factors Int Arch Occup Environ Health 2009 82 643 652 10.1007/s00420-008-0375-4 18956210 PMC2793406

[B52] Madan I Reading I Palmer KT Coggon D Cultural differences in musculoskeletal symptoms and disability Int J Epidemiol 2008 37 1181 1189 10.1093/ije/dyn085 18511493 PMC2740956

[B53] Leclerc A Niedhammer I Landre MF Ozguler A Etore P Pietri-Taleb F One-year predictive factors for various aspects of neck disorders Spine 1999 24 1455 1462 10.1097/00007632-199907150-00011 10423791

[B54] Walker-Bone K Reading I Coggon D Cooper C Palmer KT The anatomical pattern and determinants of pain in the neck and upper limbs: an epidemiologic study Pain 2004 109 45 51 10.1016/j.pain.2004.01.008 15082125

[B55] Malmusi D Artazcoz L Benach J Borrell C Perception or real illness? 2011 European Journal of Public Health: How chronic conditions contribute to gender inequalities in self-rated health 10.1093/eurpub/ckr184 22179096

[B56] Messing K Stock SR Tissot F Should studies of risk factors for musculoskeletal disorders be stratified by gender? Lessons from the 1998 Québec Health and Social Survey Scand J Work Environ Health 2009 35 96 112 10.5271/sjweh.1310 19305934

[B57] Silverstein B Fan ZJ Smith CK Bao S Howard N Spielholz P Bonauto D Viikari-Juntura E Gender adjustment or stratification in discerning upper extremity musculoskeletal disorder risk? Scand J Work Environ Health 2009 35 113 126 10.5271/sjweh.1309 19294319

[B58] Natvig B Bruusgaard D Eriksen W Localized low back pain and low back pain as part of widespread musculoskeletal pain: two different disorders? A cross-sectional population study J Rehabil Med 2001 33 21 25 10.1080/165019701300006498 11480465

[B59] Schneider S Mohnen SM Schiltenwolf M Rau C Comorbidity of low back pain: representative outcomes of a national health study in the Federal Republic of Germany Eur J Pain 2007 11 387 397 10.1016/j.ejpain.2006.05.005 16793296

[B60] Salaffi F De Angelis R Stancati A Grassi W Health-related quality of life in multiple musculoskeletal conditions: a cross-sectional population based epidemiological study II. The MAPPING study. Clin. Exp. Rheumatol 2005 23 829 839 16396701

[B61] Miranda H Kaila-Kangas L Heliövaara M Leino-Arjas P Haukka E Liira J Viikari-Juntura E Musculoskeletal pain at multiple sites and its effects on work ability in a general working population Occup Environ Med 2010 67 449 455 10.1136/oem.2009.048249 19889646

[B62] Nyman T Grooten WJA Wiktorin C Liwing J Norrman L Sickness absence and concurrent low back and neck-shoulder pain: results from the MUSIC-Norrtälje study Eur Spine J 2007 16 631 638 10.1007/s00586-006-0152-6 16741741 PMC2213552

[B63] McBeth J Symmons DP Silman AJ Allison T Webb R Brammah T Macfarlane GJ Musculoskeletal pain is associated with a long-term increased risk of cancer and cardiovascular-related mortality Rheumatology (Oxford) 2009 48 74 77 19056799 10.1093/rheumatology/ken424 PMC2639482

